# The Impairment of Osteogenesis in Bone Sialoprotein (BSP) Knockout Calvaria Cell Cultures Is Cell Density Dependent

**DOI:** 10.1371/journal.pone.0117402

**Published:** 2015-02-24

**Authors:** Guenaelle Bouet, Wafa Bouleftour, Laura Juignet, Marie-Thérèse Linossier, Mireille Thomas, Arnaud Vanden-Bossche, Jane E. Aubin, Laurence Vico, David Marchat, Luc Malaval

**Affiliations:** 1 INSERM U1059, Laboratoire de Biologie du Tissu Osseux, SFR IFRESIS, Université de Lyon and Université Jean Monnet, St-Etienne, France; 2 Dept. of Molecular Genetics, University of Toronto, Toronto, Ontario, Canada; 3 Ecole Nationale Supérieure des Mines de Saint-Etienne, Center for Health Engineering, SFR IFRESIS, Saint-Etienne, France; Institut de Génomique Fonctionnelle de Lyon, FRANCE

## Abstract

Bone sialoprotein (BSP) belongs to the "small integrin-binding ligand N-linked glycoprotein" (SIBLING) family, whose members interact with bone cells and bone mineral. BSP is strongly expressed in bone and we previously showed that BSP knockout (BSP-/-) mice have a higher bone mass than wild type (BSP+/+) littermates, with lower bone remodelling. Because baseline bone formation activity is constitutively lower in BSP-/- mice, we studied the impact of the absence of BSP on *in vitro* osteogenesis in mouse calvaria cell (MCC) cultures. MCC BSP-/- cultures exhibit fewer fibroblast (CFU-F), preosteoblast (CFU-ALP) and osteoblast colonies (bone nodules) than wild type, indicative of a lower number of osteoprogenitors. No mineralized colonies were observed in BSP-/- cultures, along with little/no expression of either osteogenic markers or SIBLING proteins MEPE or DMP1. Osteopontin (OPN) is the only SIBLING expressed in standard density BSP-/- culture, at higher levels than in wild type in early culture times. At higher plating density, the effects of the absence of BSP were partly rescued, with resumed expression of osteoblast markers and cognate SIBLING proteins, and mineralization of the mutant cultures. OPN expression and amount are further increased in high density BSP-/- cultures, while PHEX and CatB expression are differentiatlly regulated in a manner that may favor mineralization. Altogether, we found that BSP regulates mouse calvaria osteoblast cell clonogenicity, differentiation and activity in vitro in a cell density dependent manner, consistent with the effective skeletogenesis but the low levels of bone formation observed *in vivo*. The BSP knockout bone microenvironment may alter the proliferation/cell fate of early osteoprogenitors.

## Introduction

The SIBLING (Small Integrin Binding N-Linked Glycoproteins, [1]) are a family of extracellular matrix (ECM) targeted factors highly expressed in bone and dentin and which comprise BSP (Bone Sialoprotein), OPN (osteopontin), MEPE (Matrix Extracellular PhosphoglycoprotEin), DMP-1 (Dentin Matrix Protein-1) and DSPP (Dentin SialoPhosphoProtein). The SIBLING genes are aligned together in human chromosome 4 and mouse chromosome 5, in a region called the “bone gene cluster” [2][3]. These proteins all display a disordered structure and most present a large number of acidic amino acids in their sequence, favoring interactions with crystals [4]. They share multiple conserved sites such as an Arg-Gly-Asp (RGD) motif, an Acidic Serine and Aspartate Rich Motif (ASARM) and many phosphorylation sites [1]. The SIBLING play multiple and distinct roles in bone development, remodeling, healing and mineralization [1], and their specific knockouts show distinct phenotypes [5–10] All SIBLING proteins undergo important post-translational modifications, such as phosphorylation, glycosylation, sulfatation as well as proteolytic cleavage which modify/determine their functions [11] [12]. In particular, protein cleavage can generate bioactive fragments acting locally within the ECM, such as the ASARM peptide [13] [14]. The ASARM motif of MEPE is cleaved by cathepsin B (CatB) and its free form inhibits mineralization [15]. PHEX, an endopeptidase expressed mainly in bone and dentin [16] [17], binds to the ASARM motif within MEPE and prevents its release by CatB [18] [19]. PHEX was shown to bind free ASARM (from MEPE and OPN) as well, neutralize its activity through hydrolysis and thus abolish its inhibitory action on mineralization [20] [21] [13] [14]. OPN is known to be a mineralization inhibitor by binding to the apatitic mineral crystals in bone [22] [23]. Full-length, phosphorylated OPN [24] as well as its phosphorylated peptides [13] inhibit mineralization in osteoblast cultures and in vivo [25] [26]. In register with its anti-ASARM properties, PHEX was recently shown to degrade extensively full-length OPN, including the ASARM motif [27] and would thus be expected to antagonize the inhibitory activity of OPN on mineralization.

OPN and BSP are highly expressed by osteoblasts, osteoclasts and hypertrophic chondrocytes, and BSP is particularly abundant in sites of primary bone formation [28]. BSP is a potent mineralization nucleator [29] but also a matrix-associated signal promoting osteoblast differentiation as well as increased production of a mineralized matrix [30]. We previously characterized extensively adult BSP knockout (BSP-/-) mice, and showed that they have a very low bone formation activity respective to wild type (BSP+/+, [10]). Indeed, the number of osteoblastic, mineralized colonies (CFU-OB) is dramatically lower in BSP-/- bone marrow cultures [10]. Nonetheless, mutant mice display an overall normal skeleton and indeed a higher trabecular bone mass than the BSP+/+ [10], due to impaired osteoclasts recruitment and activity [31]. In the present study, we asked how normal bone could be observed after deletion of a protein which appears in vitro as a limiting factor for osteoblastogenesis/mineralized matrix production. Because of the complex cellular composition of marrow culture, we studied *in vitro* osteogenesis in cultures of calvaria cells (CC) isolated from BSP+/+ and BSP-/- six day old mice by collagenase digestion. We show in this cell culture model that BSP-/- bone cell cultures display a constitutive impairment of osteogenesis, resulting in part from a defect in osteoprogenitor numbers, and which is partly rescued by a higher cell density.

## Material and Methods

### Production of BSP-/- mice

BSP Knock-out mice were generated as described in [10]. Briefly, exons II-III of the mouse *Ibsp* gene were replaced by a PGKneo cassette that created a null allele in mouse embryonic stem (ES) cells (R1 passage 8; kindly provided by Dr. Andras Nägy [32]). After selection, positive clones were used to generate chimeric mice which were crossed to albino CD1 outbred females, and a chimeric male with germline transmission of the mutation was used to establish offspring on a 129sv/CD1 background. BSP-/- mice are viable and fertile; their phenotype has been extensively described [10] [31] [33–38].

### Care of animals

The mice were housed and bred in the PLEXAN (Platform for Experiments and Analysis) facility, Faculty of Medicine, University of Saint-Etienne, France. The procedures for the care and killing of the animals were in accordance with the European Community Standards on the care and use of laboratory animals (Ministère de l'Agriculture, France, Authorization 04827). All animal experiments were approved by the "Comité du Bien-Etre Animal" (Animal Welfare Committee) of the PLEXAN. Mice were kept at standard temperature (23±2°C), in a light controlled environment (12h light/12h dark cycle), were fed a standard pellet diet (A03 food, Scientific Animal Food and Engineering, Aury, France) and were allowed water ad libitum. For bone marrow cultures, 2 months old mice were killed by cervical dislocation, and their long bones were dissected out. For calvaria cell preparation, five days old mice were killed by decapitation, and their skull caps (calvarium) were dissected out. Bone samples were immediately processed in sterile conditions, as described below.

### Cell isolation and culture

Bone marrow cells were flushed-out from cleaned long bones in culture medium, using a syringe fitted with a 21G needle, and grown in a T75 flask for one week. Cells were then trypsinized, counted and plated at 5000/cm^2^ in 96 well plates for MTT assay and in T25 flasks for ALP-positive colony counting. Primary mouse calvaria cells (MCC) were isolated from skull bones of 15 to 30 neonatal (5 days old) mice (from 3 to 6 litters) by an enzymatic digestion process. The calvaria were dissected-out, and all connective tissues were carefully removed. The samples were cleaned, cut in two halves along the sagittal suture and pooled [39]. The fragments were then digested at 37°C in 0.4% type I collagenase (Sigma, Saint-Quentin Fallavier, France) in Ham's *F-12* Nutrient Mixture (Sigma), under gentle stirring. The first 5 min digest was discarded. Six consecutive extractions (5 to 20 min) were performed. After centrifugation, calvaria cells were collected and plated in T-75 flasks in minimum essential medium eagle (Sigma) containing 2 mM L-glutamine (Sigma), 50 U/ml penicillin (Sigma), 50 μg/ml streptomycin (Sigma), and 10% fetal calf serum (FCS), hereafter referred to as “culture medium”. After 24h, the cells were trypsinized and replated at very low density (50 cells/cm^2^) for isolated colonies assay, or at standard (5 000 cells/cm^2^), or high density (25 000 cells/cm^2^) for dense cultures.

To induce osteogenesis, the culture medium was supplemented with 50 μg/ml ascorbic acid (Sigma) and 10 mM beta-glycerophosphate (Sigma). Cell culture was performed at 37°C under 5% CO_2_. The medium was changed every two or three days. In some cultures, a specific cathepsin B inhibitor (CA074, CAS #: 134448–10–5, Sigma), was added continuously or by time windows to the culture medium, at doses ranging from 1 to 100 μg/ml. In other experiments, 0.5μg/ml each of leupeptin (CAS #: 103476–89–7, Sigma) and pestatin A (CAS #: 26305–03–3, Sigma) was added to the culture starting at day (D) 2. Arrested cultures were fixed with buffered PFA, or extracted with appropriate medium for QRT-PCR or protein analysis (see below). Mineralized colonies were stained with the Von Kossa procedure. Labeling for ALP-positive cells was performed according to the manufacturer's instruction (kit #: 85L2, Sigma).

### Colony assays

In counting colony forming units (CFU = single progenitors identified by a colony containing their cellular progeny) at low density, we distinguished between colonies whose cells did not express ALP (fibroblasts = CFU-F) and those containing ALP-expressing cells (CFU-ALP, committed to the osteoblast lineage but not yet mineralized). Von-Kossa stained, i.e. mineralized osteoblast colonies (CFU-OB) were counted in standard and high density cultures.

### Cell attachment assay

Thirty minutes, 1 hour and 2 hours after plating, calvaria cells were washed twice with PBS to rinse off unattached cells, fixed with 3.7% PFA and stained with 4&rsquo;6-diamidino-2-phenylindole (DAPI, Chemicon, Temecula, CA, 0,1μg/ml). Cells were examined with a Zeiss AxioObserver fluorescent videomicroscope (Zeiss, Le Pecq, France) and images acquired with the AxioCam camera using the AxioVision software (Zeiss, version 4.8.1.0). The nuclei were then counted using the Image J software (http://rsb.info.nih.gov/ij).

### Growth, proliferation and apoptosis assays

Cell growth curves from day 1 to day 13 were established using the MTT (3-(4,5-Dimethylthiazol-2-yl)-2,5-diphenyltetrazolium bromide) viability assay (Sigma). The cells were incubated (37°C, 5% CO2) with 0.5 mg/ml MTT for 4hr, then DMSO (Sigma) was added for crystal solubilisation before reading the optical density (OD) of the product at 540nm. The linearity of formazan production was verified with cells plated at known densities (not shown). To assay for proliferation, two techniques were used. Calvaria cells were grown on glass coverslips (10mm diameter) in multiwell plates and fixed at D1, D2 and D6, then permeabilized and immunolabeled for the proliferation marker Ki67 (Sigma) using the horseradish peroxidase Vectastain kit (AbCys, Paris, France) and a DAB substrate kit (AbCys), according to the manufacturer’s instructions. Ki67 positive and negative nuclei were then counted in 10 fields per coverslips (n = 6 coverslips/genotype/time-point) using Image J. To take into account the difference in growth kinetics, the data were normalized to 1000 DAPI-labelled nuclei in the bar graph. In other dishes, a colorimetric immunoassay was used based on the measurement of BrdU (pyrimidine analogue 5-bromo-2’-deoxyuridine) incorporation (Cell proliferation ELISA BrdU kit, Roche Diagnostic GmbH, Mannheim, Germany). Apoptosis was assayed by fluorescent transferase-mediated dUTP nick-end labeling (TUNEL) assay using the ApoTag PLUS Fluorescein In Situ Apoptosis Detection kit (Serologicals, Norcross, GA, USA). As a positive control, some wells were incubated with 10^–8^ M dexamethasone overnight prior to the assay.

### Messenger RNA extraction and quantitative real-time polymerase chain reaction (QRT-PCR)

Total RNA was extracted from calvaria cell cultures grown for 3, 6, 14 and 17 days, using TRI Reagent (Sigma) according to the manufacturer’s instructions. Briefly, the extracts were centrifuged at 10 000g for 10 min at 4°C to remove cell debris. Chloroform was added to separate the aqueous phase containing RNA from the interphase and the organic phase. The aqueous phase was recovered and precipitated with isopropanol. The RNA pellets were then washed in 70% ethanol in RNase-free water. Finally, after air-drying of 1–2 min, purified RNA pellets were dissolved in RNase-free water and RNA concentration was assessed with the Ribogreen kit (Invitrogen, Life Technologies, Eugene, OR, USA). RNA quality was checked with the Experion automated electrophoresis station (BIO-RAD, Hercules, CA, USA). Samples were stored at -80°C until used. Complementary DNA (cDNA) was synthesized from 1 μg of total RNA with the iScript cDNA Synthesis kit for Thermocycler (BIO-RAD) according to the manufacturer’s instruction. For QRT-PCR, 0.04μg of cDNA mixture were prepared for CFX96TM (BIO-RAD), real time PCR detection system, using SYBR Green I dye (Lightcycler faststart DNA master SYBR green I, Roche). Primer sequences and full names of the markers are listed in Table 1. For each gene, expression levels were normalized to cyclophilin A, whose expression did not change during the culture time-course in the conditions used (not shown). Amplified product sizes were checked on a 2% agarose gel with 1 μg/ml ethidium bromide and DNA molecular weight marker.

**Table 1:** Oligonucleotides sequences used in QRT-PCR.

Genes	Forward	Reverse	Product size (pb)	Source
OCN	ctctgacctcacagatgccaa	ctggtctgatagctcgtcaca	190	NM_007541.2
Col1α1	caccctcaagagcctgagtc	ttaggcgcaggaaggtcagc	374	NM_007742.3
ALP	agttactggcgacagcaagc	ggacctgagcgttggtgtta	244	NM_007431.2
OSX	atggcgtcctctctgcttg	aaggtcagcgtatggcttct	153	NM_130458.3
Runx2	ccgggaatgatgagaactac	tgtctgtgccttcttggttc	223	NM_009820.4
OPN	cccggtgaaagtgactgattc	atggctttcattggaattgc	193	NM_009263.2
BSP	cggaggagacaacggagaag	gtaagtgtcgccacgaggct	295	NM_008318.3
DMP1	ttcgagaacttcgctgaggt	ttgtggtatctggcaactgg	135	NM_016779.2
MEPE	aggctgtgtctgttggactg	ccatcctctgtgccttcatc	157	NM_053172.2
PHEX	agaattctcaagggtaatccaggg	tgcactctcaataaagttgacacattt	79	NM_011077.2
CatB	gcgtgtctaacagtgtgaag	ccagtcaaggttccaagagt	221	NM_007798.2
Cyclo A	tacacgccataatggcactg	ccatggcttccacaatgttc	227	NM_008907.1

*Abbreviations*: OCN: osteocalcin; Col1α1: collagen 1 alpha 1 chain; ALP: alkaline phosphatase; OSX: osterix; Runx2: runt-related transcription factor 2; OPN: osteopontin; BSP: bone sialoprotein; DMP1: dentin matrix protein 1; MEPE: matrix extracellular phosphoglycoprotein; PHEX: phosphate regulating endopeptidase homolog, X-linked; CatB: cathepsin B; Cyclo A: cyclophillin A.

### Immunolabelling

For immunolabeling, cells were grown on 10-mm-diameter glass coverslips for 10 days, then fixed with 3.7% PFA and permeabilized in methanol. Cultures were labeled overnight at 4°C in a humid chamber with an anti-mouse OPN primary monoclonal antibody (Santa Cruz, Dallas, USA, ref sc-21742) diluted 1:100 in PBS with 10% goat serum (labeling buffer). After washes in PBS, the cells were incubated for 1.5 hour at room temperature with a mixture of Alexa 488–labeled goat anti-mouse secondary antibody and 4&rsquo;6-diamidino-2-phenylindole (DAPI, Chemicon, Temecula, CA, 1 μg/ml) in labeling buffer. After rinsing, cells were examined with a Zeiss AxioObserver fluorescent videomicroscope and images acquired with the AxioCam camera using the AxioVision software.

### Osteopontin immunoassay

After 6, 14 and 17 days of culture, ELISA was performed to measure OPN in cell lysate and culture medium using the DuoSet mouse Osteopontin ELISA Development System (R&D Systems, Minneapolis, MN, USA) according to the manufacturer’s instructions. Cells were collected in a lysis buffer composed of PBS with 0.5% Nonidet 40 and 1% protease inhibition cocktail (PIC, Sigma), 1μl/ml triton (Sigma) and 1,8 mg/ml iodometacin (Sigma). Each sample was run in duplicate in the assays. Data were corrected for fresh culture medium or fresh lysis buffer values and normalized to total protein content of the cell lysate measured with the BCA Protein Assay Kit (Interchim Pierce, Monluçon, France).

### Statistical analysis

The STATISTICA software (version 8.2; StataCorp, College Station, TX) was used for statistical analysis. Quantitative data are presented as mean±SD (except for Ki67 labeling, as mentioned), from one experiments in a series of two to three repeats, done with distinct cell isolates and giving the same results. Inter-group differences were tested with the non-parametric Mann-Whitney U-test for all experiments except Ki67 labeling. For the latter, the total numbers of positive and negative cells for each day in each genotype were plotted in a contingency table and assessed for homogeneity with a χ_2_ test.

## Results

### Clonogenicity and osteoblast differentiation in BSP-/- bone marrow and MCC cultures

**Fig 1:**
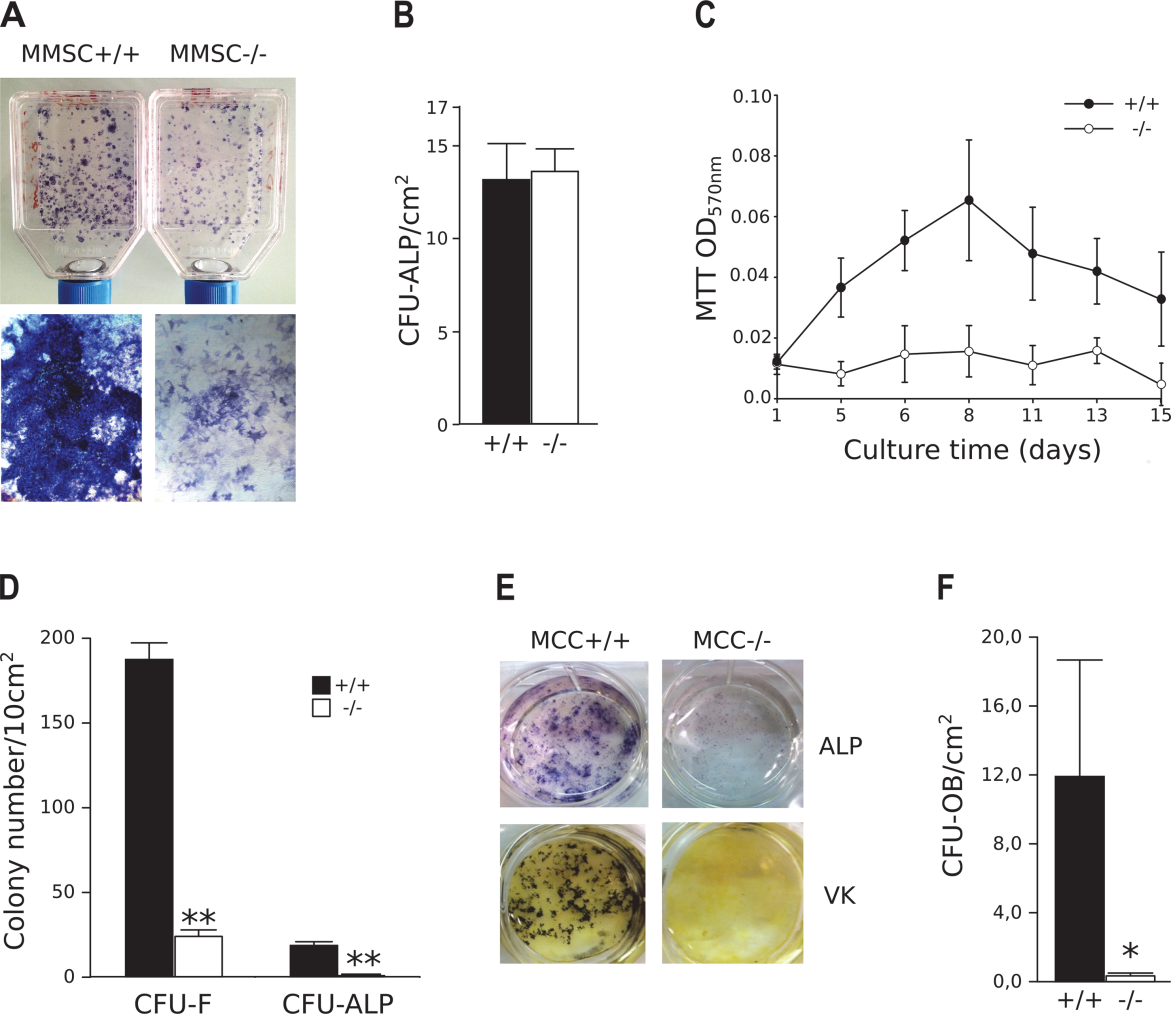
*In vitro* osteoblast differentiation in mouse bone marrow stromal cell (A-C) and mouse calvaria cell (MCC, D-F) cultures. (A) micrographs and (B) quantification of unmineralized, ALP+ colonies (CFU-ALP) in bone marrow stromal cell cultures (D12) from BSP-/- and +/+ mice. N = 13 dishes/genotype. (C) Time-course of *in vitro* cell growth in BSP+/+ and BSP-/- bone marrow cultures. N = 12 wells/time point. (D) Quantification of total colony forming units-fibroblasts (CFU-F) and CFU-ALP in low density cultures (50 cells/cm^2^) of BSP+/+ and BSP-/- MCC at D15. N = 5 dishes/group. (E) Micrographs of ALP+ and Von Kossa (VK) stained standard density (5000 cells/cm^2^) MCC cultures at D20. (F) Quantification of mineralized colonies (CFU-OB) in standard density BSP-/- and BSP+/+ MCC cultures at D20. N = 3 dishes/group. *: p<0.05, **: p< 0.01 vs BSP+/+; Mann-Whitney U Test.

As previously published [10], the number of progenitors (CFU) giving birth to colonies with ALP+ cells but not yet mineralized matrix (CFU-ALP) was the same in BSP-/- and BSP+/+ bone marrow cultures (Fig. 1A, B). However, these colonies were clearly smaller in mutant cultures (Fig. 1A) and the MTT assay showed that their growth was blunted compared to the BSP+/+ (Fig. 1C). CFU-F and CFU-ALP numbers were assessed in BSP+/+ and-/- MCC cultures plated at low density. In contrast to marrow cultures, the numbers of CFU-F and CFU-ALP formed were lower in BSP-/- MCC cultures than in BSP+/+ (Fig. 1D), and the size of CFU-ALP colonies was also lower than in BSP+/+ in low (not shown), as in standard density cultures (Fig. 1E). We used low density MCC cultures to facilitate colony counting in the dishes, although mineralized osteoblast colonies (CFU-OB), also known as "bone nodules" are not numerous at low density and their appearance is delayed [40]. We counted the CFU-OB at standard plating density, and as expected we found that very few formed in mutant culture dishes (Fig. 1E, F).

### Growth, attachment, proliferation and apoptosis in calvaria cell cultures from BSP-/- and +/+ mice

**Fig 2:**
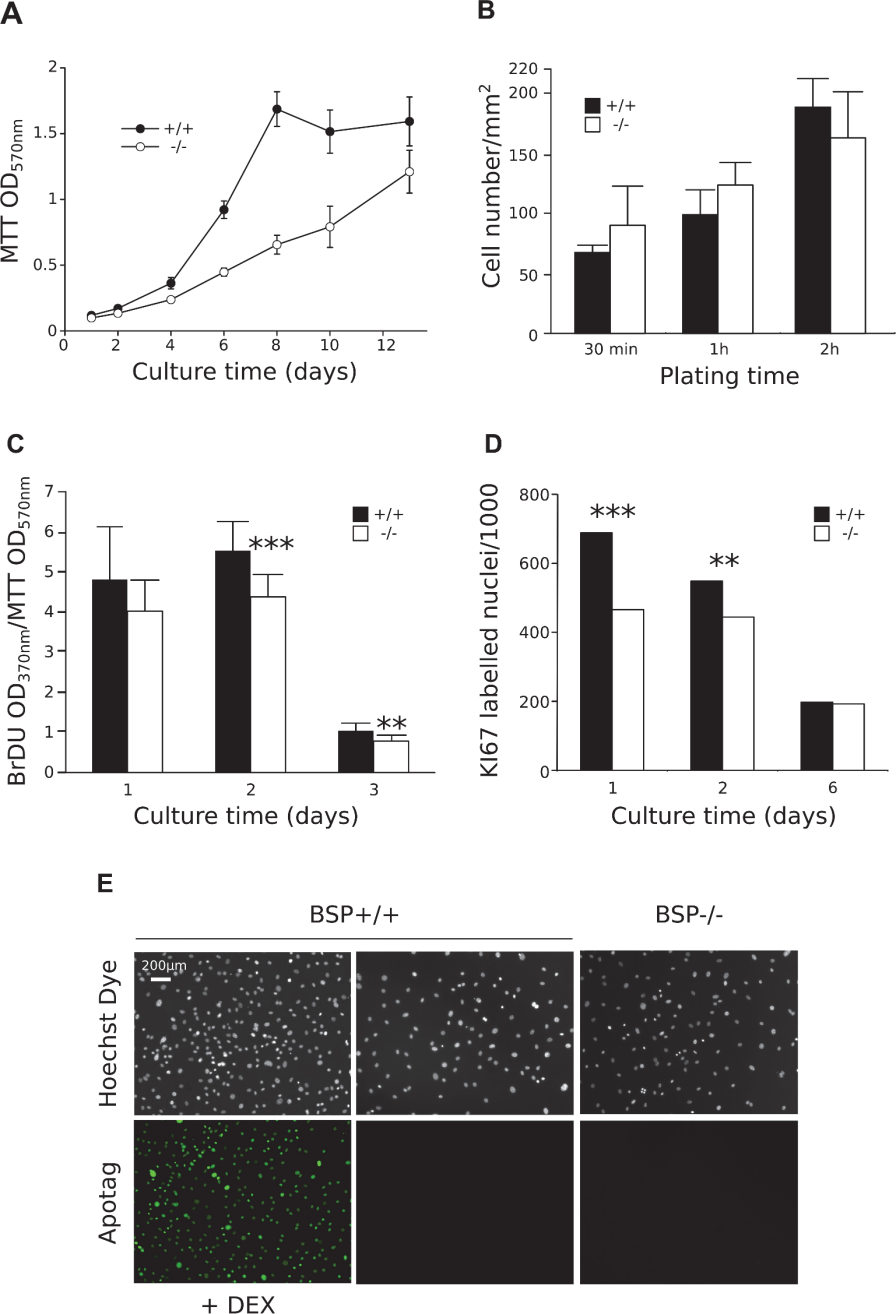
Growth, attachment, proliferation and apoptosis in standard density calvaria cell cultures of BSP-/- and BSP+/+ mice. (A) Time-course of *in vitro* cell growth in BSP+/+ and BSP-/- MCC cultures N = 12 dishes/time point. (B) Number of adherent BSP-/- and BSP+/+ MCC 30 min, 1 hr and 2 hrs after plating. N = 4 dishes/group. (C and D) *In vitro* proliferation in BSP+/+ and BSP-/- MCC cultures assayed by BrdU incorporation (C, N = 12 dishes/group) and KI67 labelling (D, N = 6 coverslips/genotype/time-point, 10 fields analysed/coverslip, results expressed as number of labelled cells/1000). (E) *In vitro* apoptosis in BSP+/+ and BSP-/- MCC cultures assayed by TUNEL at D6. **: p< 0.01, ***: p< 0.001 vs BSP+/+; Mann-Whitney U Test and (D) contingency table with χ_2_ test.

MTT assay of MCC cultures showed a delayed growth of BSP-/- cells (Fig. 2A). While the rate of MCC attachment was the same for both genotypes (Fig. 2B), both BrDU incorporation rate (Fig. 2C) and Ki67 labelling (Fig. 2D) showed that BSP-/- MCC cultures exhibit a lower proliferation rate at early culture times and until D6, time at which no difference in proliferation was observed between the two genotypes (Fig. 2D). While high numbers of dexamethasone-induced apoptotic cells were detected in the positive control, no significant levels of apoptosis were observed in BSP+/+ or BSP-/- MCC cultures when assayed at D6 with the TUNEL methods (Fig. 2E).

### Time-course of osteoblast-associated and SIBLING protein expression in osteogenic MCC cultures

**Fig 3:**
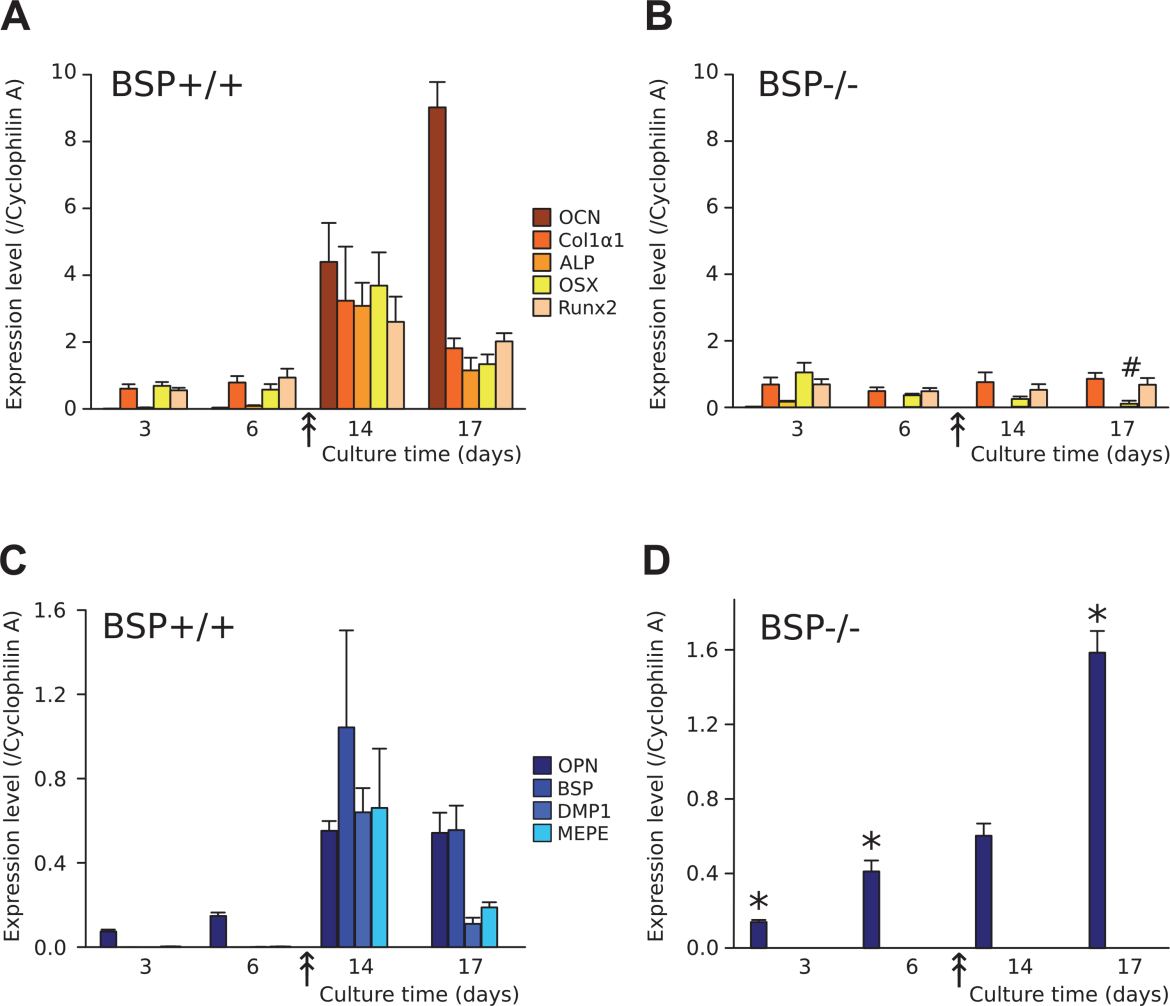
Effects of BSP deletion on the time-course of osteoblast marker and SIBLING expression in standard density MCC cultures. (A-D) Time-course of expression of osteoblast-associated gene: osteocalcin (OCN), type 1 collagen (Col1α1), alkaline phosphatase (ALP), osterix (OSX) and Runx2 and SIBLING genes: osteopontin (OPN), bone sialoprotein (BSP), DMP1 and MEPE at successive time-points of BSP+/+ (A, C) and BSP-/- (B, D) MCC cultures. The arrow marks the appearance of bone nodules. N = 3 dishes/group. *: p< 0.05 vs time-matched BSP+/+; #: p<0.05 vs D3 to D14. Mann-Whitney U Test.

**Fig 4:**
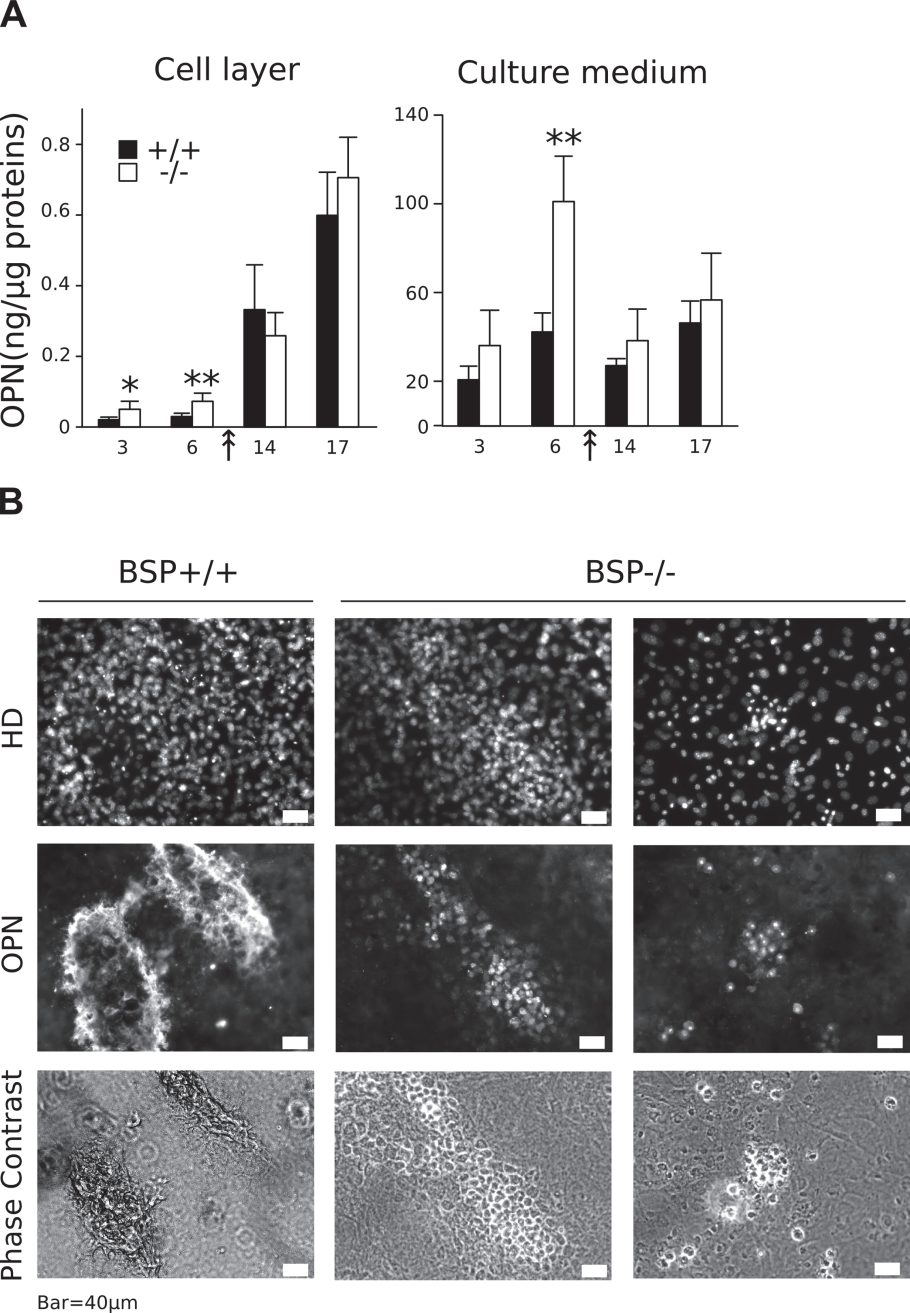
Expression of OPN protein in standard density MCC cultures. (A) OPN amounts were assayed in the cell layer and culture medium of BSP+/+ and BSP-/- MCC cultures at D3, 6, 14 and 17. Results are normalized on total cell layer proteins. N = 6 dishes/time point. *: p<0.05, **: p<0.01 vs time-matched BSP+/+, Mann-Whitney U Test. (B) BSP+/+ and BSP-/- MCC cultures were arrested at D10 and immunolabeled for OPN. Nuclei were labeled with DAPI and the same fields were imaged in phase contrast microscopy. White bar = 40μm.

The kinetics of proliferation and osteoblast differentiation in rodent calvaria cultures has been abundantly described [39–41]. After a phase of fast culture growth, bone nodules appear in the culture when the cells have reached confluence; they progressively increase in number and size, and mineralize. In our BSP+/+ cultures, confluence occurred around day 10 (Fig. 2A) and the first nodules were visible by that time (indicated by the double arrow in Fig. 3 and 4).

The expression of osteoblast-associated genes was analysed between D3 and D17 of BSP+/+ and BSP-/- MCC culture plated at standard density. Expression levels of most markers assayed were not different between the two genotypes at D3 and D6 (Fig. 3A, B). Starting at D14, in coincidence with the appearance of CFU-OB in the cultures, osteoblastic genes ALP, Coll1α1, OSX, Runx2 and the specific mature osteoblast gene OCN were highly expressed in BSP+/+ MCC (Fig. 3A). However, in BSP-/- MCC cultures their expression levels remained the same as at earlier time-points, except for OSX whose levels decreased significantly in late cultures (Fig. 3B). Similarly, most SIBLING mRNA were highly expressed in BSP+/+ MCC starting at D14, with earlier expression of OPN at D3 and D6 (Fig. 3C). Strikingly, no significant expression of DMP1 nor MEPE was observed in BSP-/- MCC standard-density cultures at any time-point (D3, D6, Fig. 3D). On the other hand, OPN mRNA was overexpressed in early and late (D17) cultures respective to BSP+/+ cells (Fig. 3C, D).

OPN protein levels in the cell layer increased progressively after day 6, with values still higher than wild type at early time points in mutant cultures (Fig. 4A). OPN in the culture medium remained stable at all time points when normalized on cell layer protein amounts, except for a peak of overexpression in BSP-/- cultures vs BSP+/+ at day 6 (Fig. 4A); of note, the graph of absolute values in the culture medium showed a similar pattern, with additional overexpression at day 3 in BSP-/- cultures (not shown). Immunolabelling of BSP+/+ MCC cultures at day 10 (i.e. about the time of appearance of bone nodules) shows that OPN protein is mostly detected in the matrix of bone colonies, colocalizing with incipient mineralization, as witnessed by phase-contrast images (Fig. 4B). In BSP-/- cultures, most colonies comprise very few cells (Fig. 4B, right column), although scant significant sized colonies can be found (Fig. 4B, middle column). In all BSP-/- colonies, labelling for OPN is restricted to cell cytoplasm, and does not concern the extracellular matrix, which remains unmineralized.

### Mineralization in high density BSP-/- MCC cultures

**Fig 5:**
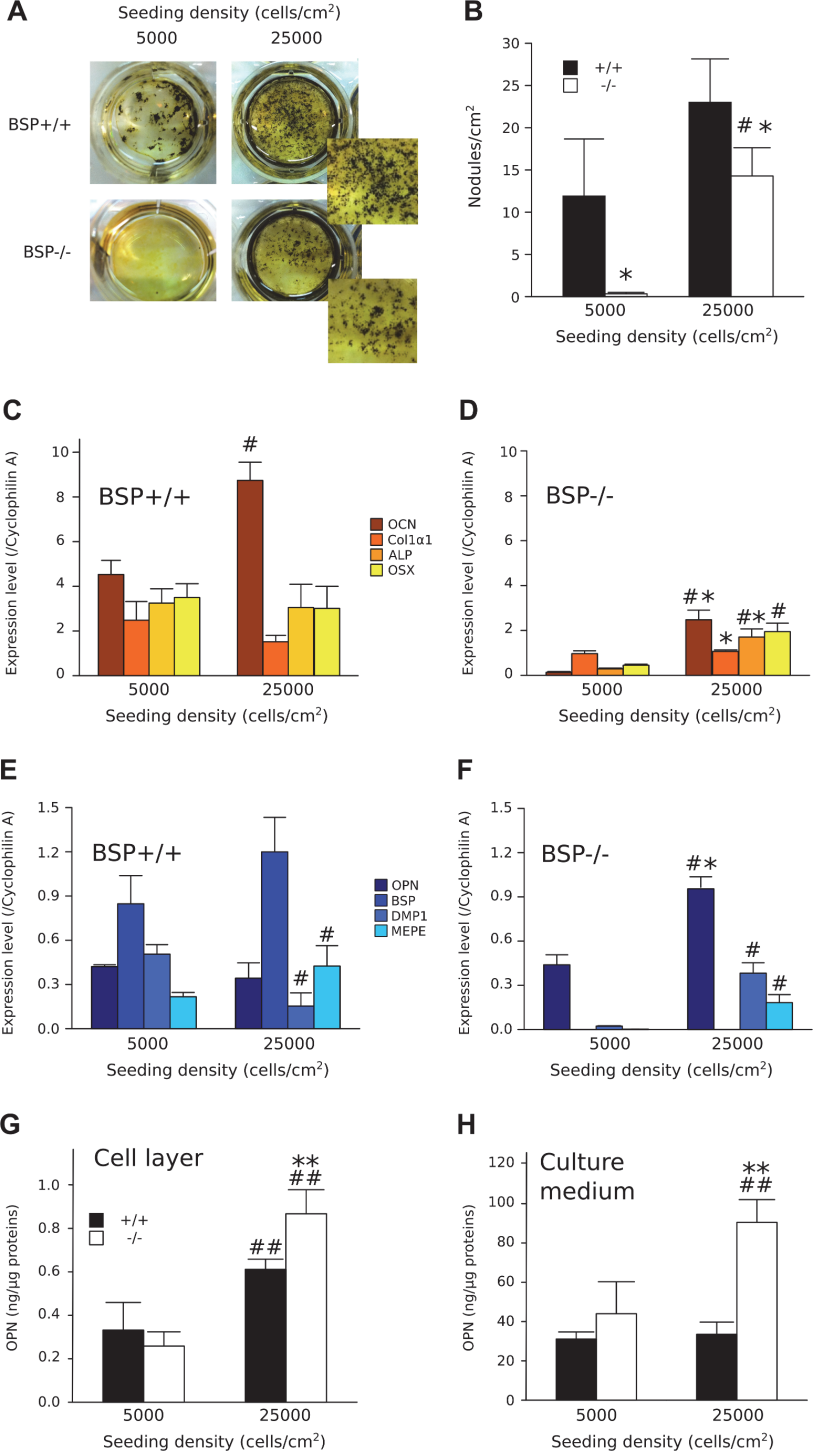
*In vitro* osteoblast differentiation in standard and high density BSP+/+ and BSP-/- MCC cultures. (A) Micrographs (Insets: higher magnification) and (B) quantification of total mineralized colonies formed at D14 in BSP+/+ and BSP-/- MCC cultures grown at standard (5000 cells/cm^2^) and high density (25000 cells/cm^2^). N = 3 dishes/group. Expression of osteoblast-associated (C and D) and SIBLING genes (E and F) at D14 in BSP+/+ (C, E) and BSP-/- (D, F) cultures. N = 3 dishes/group. Quantification of OPN in the cell layer (G) and the culture medium (H) of BSP+/+ and BSP-/- standard and high density cultures. Results are normalized on total cell layer proteins. N = 6 dishes/group. *: p< 0.05, **: p<0.01 vs BSP+/+; #: p< 0.05, ##: p<0.01 vs standard density. Mann-Whitney U Test.

In contrast to low and standards density plating, high density (25000 cell/cm^2^) BSP-/- MCC cultures did form mineralized colonies at day 14, although smaller and in lower numbers (Fig. 5A, B). In the BSP+/+, the expression levels of osteoblast markers did not change between the two conditions, except for increased OCN in high density cultures (Fig. 5C). In contrast, expression of OCN, ALP and OSX increased significantly in BSP-/- high density cultures, although most markers remained below BSP+/+ values (Fig. 5D). A similar pattern was observed with SIBLING protein expression (Fig. 5E, F). High density culture of BSP+/+ cells did not significantly affect OPN nor BSP expression levels. In contrast, DMP1 expression was decreased, while MEPE expression was increased at high density (Fig. 5E). The mRNA levels of OPN were increased and expression of DMP1 and MEPE became significant in BSP-/- high density cultures compared to standard (Fig. 5F). OPN protein levels in BSP+/+ and BSP-/- cell layers were higher in high density cultures (Fig. 5G); values were also higher than standard density in the culture medium of BSP-/-, but not BSP+/+ dense cultures (Fig. 5H).

### Effects of plating density on PHEX and Cathepsin B expression in BSP+/+ and BSP-/- MCC cultures

**Fig 6:**
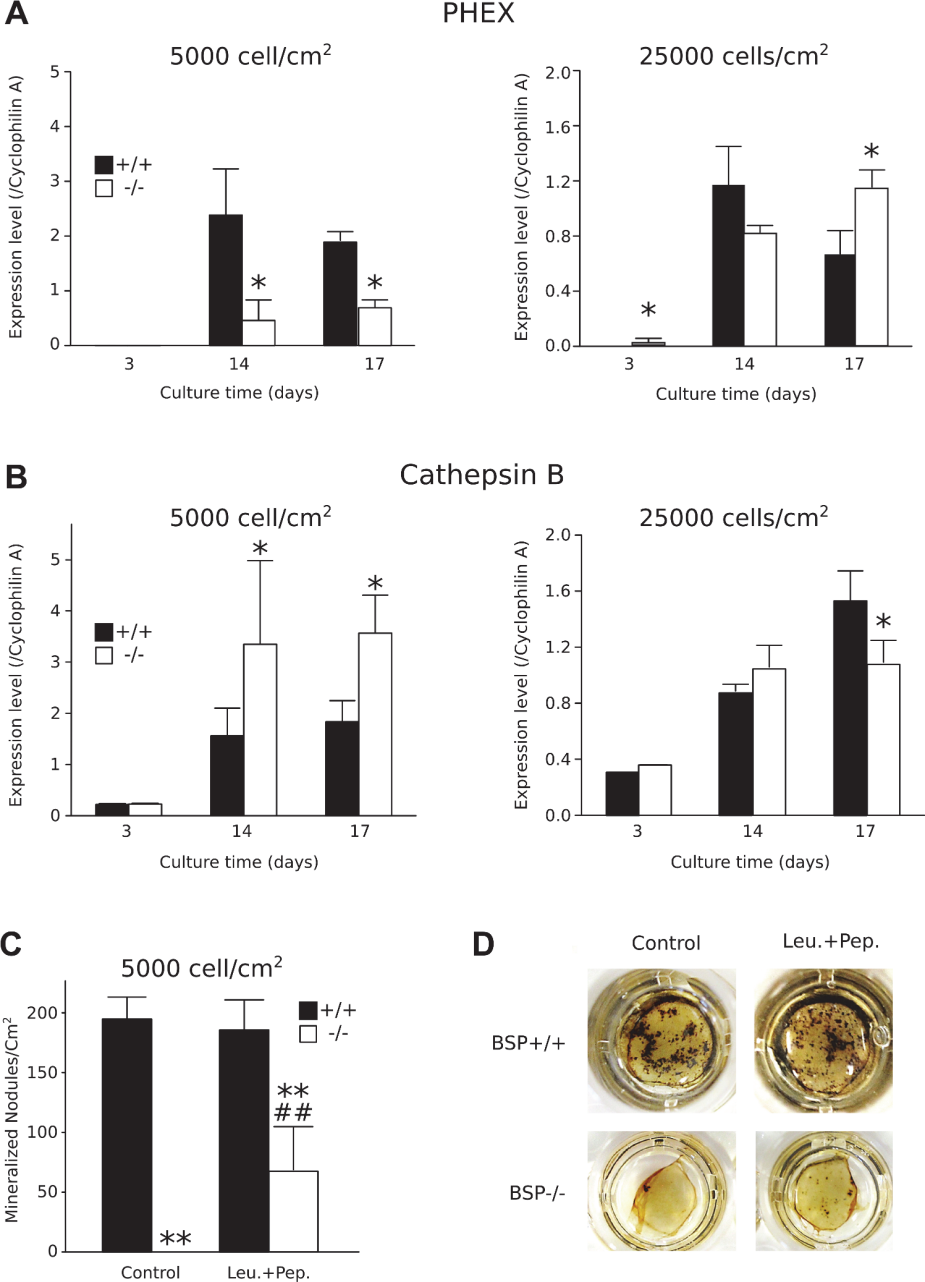
Time-course expression of PHEX (A) and cathepsin B (CatB, B) in BSP+/+ and-/- MCC cultures plated at standard and high density. N = 3 dishes/group. * p< 0.05 vs time-matched BSP+/+. (C) Quantification and (D) micrography of mineralized colonies at D17 in standard BSP-/- and BSP+/+ MCC cultures treated from D2 to D17 with 1μg/ml protease inhibitor mix (Leu.+Pep. = leupeptin + pepstatin A). N = 6 dishes/group. **: p< 0.01 vs genotype-matched control; ##: p<0.01 vs BSP+/+. Mann-Whitney U Test.

PHEX and CatB were highly expressed in MCC cultures of both genotypes starting at D14 (Fig. 6A, B). However, at standard density, PHEX expression was lower in BSP-/- dishes than in BSP+/+ in mature cultures (D14 and D17), while CatB expression was higher. In high density BSP+/+ cultures, expression of both enzymes decreased significantly (Fig. 6A, B). In high-density BSP-/- dishes, PHEX expression was increased respective to standard density, while expression of CatB was decreased. As a result, expression of both enzymes in mutant cultures reached the same levels as in wild type at D14, and PHEX was higher and CathB lower than in BSP+/+ at D17 (Fig. 6A, B). Blocking CatB activity by treating low density BSP+/+ and BSP-/- MCC cultures with a specific inhibitor, CA074, did not significantly affect nodule production nor mineralization in either genotypes (results not shown). BSP-/- MCC cultures treated with a mix of inhibitors (leupeptine+pepstatine) targeting a broad range of proteases presented significantly more mineralized colonies compared to untreated BSP-/- cells (Fig. 6C, D).

## Discussion

Although BSP has long been considered a marker of late osteoblastic differentiation, the actual role(s) of the protein in bone biology remain poorly known. Previous studies have shown that BSP directly impacts the differentiation of osteoblasts [30]. BSP overexpression increases osteoblast-related gene expression and enhances mineralized nodule formation in culture. Conversely, the reduction of BSP expression in osteoblasts with specific shRNA inhibits the expression of osteoblast markers, leading to a significantly reduced matrix mineralization [30]. Taken together, these results indicate that BSP might serve as a matrix-associated signal regulating osteoblast differentiation and mineralized matrix production.

In the present study, we have shown that BSP-/- MCC attach normally to the substrate, but display an impaired clonogenicity and differentiation. They exhibit delayed growth with a lower proliferation rate at early culture times, later normalized. Along with the lower number of CFU-F counted in BSP-/- MCC, these results suggest that less progenitors (= proliferating cells) were seeded at the start of the BSP-/- cultures, rather than any intrinsic difference in the culture proliferative rate between the two genotypes, or differential levels of apoptosis, which appeared negligible in these cultures. Earlier work in rat suggested the existence of early, glucocorticoid dependant, ALP-negative progenitors present in the bone marrow and of late, glucocorticoid independant ALP+ progenitors dominant in the calvaria cell population [42–44]. If this applies to mice, MCC progenitors would be more advanced stages than those found in the bone marrow. These advanced progenitors would logically be in lower number if the progeny of earlier cell stages is reduced in BSP-/- mutants, as witnessed by the smaller CFU-ALP in bone marrow cultures. Thus, a BSP-/- bone microenvironment would alter the proliferation potential and/or cell fate of early osteoprogenitors.

The impaired differentiation and mineralization observed in BSP-/- cultures is reflected in the absence of a surge in the expression of osteoblast-associated genes during the osteogenesis phase. Among the SIBLING family genes only OPN is significantly expressed in BSP-/- MCC cultures, and consistently higher than in BSP+/+ in terms of both mRNA and protein at early time points (i.e. before the appearance of bone nodules). The immunolabeling of OPN in BSP-/- colonies is restricted to the cellular cytoplasm, where it is much higher than in the BSP+/+. This is congruent with both OPN overexpression in mutant cultures and the absence of the mineralized matrix which concentrates it in the wild type. The relative overabundance of OPN in the culture medium of mutant MCC around the confluence stage (D6), when secreted OPN would begin to be trapped in the matrix of incipient nodules, might reflect both the overexpression of the protein and its preferential release in the serum. At later time-points, protease expression and thus OPN degradation might be higher, as suggested by our results (Fig. 6) and blunt the difference. Of note, QRT-PCR results (Fig. 3) and protein data (e.g. ELISA, D17, Fig. 4) are not directly comparable, because of the different normalization procedures, the distinct time-frames (instant mRNA amount vs accumulated proteins) and the prominent enzymatic processing of OPN (see below). We have previously shown that the BSP-/- phenotype is associated with lifelong overexpression of OPN [37]. This upregulation might be in part compensatory. We have also shown that the expression of either BSP or OPN is required for the anabolic response of mouse calvaria bones to PTH injection, as this response is severely blunted after extinction of OPN in BSP-/- mice, suggesting that the functions of these two proteins partly overlap [38]. Nonetheless, in MCC cultures OPN overexpression is obviously not enough to compensate for the absence of BSP at least at standard density, and the role of each SIBLING in bone formation and matrix mineralization remains to be clarified.

Our *in vitro* results at low (50 cells/cm^2^) and standard (5000 cells/cm^2^) plating density are not consistent with the *in vivo* situation of BSP-/- mice, in which we observed a globally normal skeletal development and normal bone forming activity [10], even though fetal/newborn skull and long bone matrix are undermineralized [10][37]. Therefore, we asked whether cell density could affect the development of the osteoblast phenotype in the absence of BSP. Indeed, bone marker and SIBLING gene expression increase in high density (25000 cells/cm^2^) BSP-/- cultures. Moreover, numerous mineralized nodules do form in the dishes, although they are smaller and in lower number than in BSP+/+. Therefore, the effects of the mutation appear to be at least partly compensated at high cell density. Cell confluence is a major rate-limiting factor for acid ascorbic-induced osteoblast differentiation and a recent study showed that cell-cell interactions through cadherins mediate osteoblast differentiation through up-regulation of the transcription factor EB1 [45].

While it is possible that BSP would play a part in the density-dependent differentiation of osteoblasts, how exactly osteoblast differentiation and mineralization are inhibited in standard density BSP-/- MCC cultures is still an open question. MEPE ASARM is cleaved-out and freed by CatB, and captured/degraded by PHEX. In BSP-/- standard density cultures PHEX expression is lower and CatB is higher as compared to BSP+/+, which may result in higher amounts of active, inhibitory ASARM. However, neither MEPE nor DMP1 are expressed in these cultures, and the only possible source of ASARM is OPN. In high density BSP-/- cultures, DMP1 and MEPE are expressed, but PHEX expression increases and CatB decreases, suggesting reduced ASARM peptide amounts and a lesser inhibition of mineralization. Blocking CatB activity in our standard density cultures continuously or within time-windows and with different concentration of CU074, a specific inhibitor, did not affect nodule formation or mineralization in either genotype, suggesting that this protease is not involved. There is presently no evidence that CatB is the enzyme cleaving out the ASARM from OPN, and other enzymes should be sought for. In BSP-/- MCC cultures, we used a cocktail of two inhibitors targeting a wide spectrum of protease activities, including cathepsins D and B. This treatment induced an increase of the number of mineralized colonies formed in the dishes. While this suggests that proteases are at least in part involved in the inhibition mechanism, it is not established at present that the inhibitor treatment targeted an enzyme cleaving OPN by the ASARM peptide or at any other point in the sequence, and as mentioned both full-length OPN and some of its peptides appear able to block mineralization [13]. Further studies will be therefore required to assess the involvement of OPN and/or its ASARM peptide in the inhibition of mineralization of BSP-/- MCC cultures.

In conclusion, the present study shows that BSP regulates mouse calvaria osteoblast cell clonogenicity, differentiation and activity *in vitro*, consistent with low levels of bone forming activity *in vivo*. The BSP knockout bone microenvironment may alter the proliferation/cell fate of early osteoprogenitors, explaining the smaller size of the CFU-ALP observed in bone marrow cultures and the lower number of CFU in MCC cultures. The proteolytic processing of the OPN protein might play a part in the inhibition of osteogenesis and mineralization in the absence of BSP. These hypotheses will orient future studies aimed at clarifying the respective roles of SIBLING proteins on osteogenesis.
